# Epitope-Specific Humoral Responses to Human Cytomegalovirus Glycoprotein-B Vaccine With MF59: Anti-AD2 Levels Correlate With Protection From Viremia

**DOI:** 10.1093/infdis/jiy102

**Published:** 2018-03-08

**Authors:** Ilona Baraniak, Barbara Kropff, Gary R McLean, Sylvie Pichon, Fabienne Piras-Douce, Richard S B Milne, Colette Smith, Michael Mach, Paul D Griffiths, Matthew B Reeves

**Affiliations:** 1 Institute for Immunity and Transplantation, University College London, United Kingdom; 2 Institut für Klinische und Molekulare Virologie, Friedrich-Alexander-Universität Erlangen-Nürnberg, Germany; 3 Cellular and Molecular Immunology Research Centre, London Metropolitan University, United Kingdom; 4 Clinical Development, Sanofi Pasteur, Marcy l’Etoile, France; 5 Research Department of Infection and Population Health, University College London, United Kingdom

**Keywords:** HCMV, vaccine, humoral responses, AD2, glycoprotein B

## Abstract

The human cytomegalovirus (HCMV) virion envelope protein glycoprotein B (gB) is essential for viral entry and represents a major target for humoral responses following infection. Previously, a phase 2 placebo-controlled clinical trial conducted in solid organ transplant candidates demonstrated that vaccination with gB plus MF59 adjuvant significantly increased gB enzyme-linked immunosorbent assay (ELISA) antibody levels whose titer correlated directly with protection against posttransplant viremia. The aim of the current study was to investigate in more detail this protective humoral response in vaccinated seropositive transplant recipients. We focused on 4 key antigenic domains (AD) of gB (AD1, AD2, AD4, and AD5), measuring antibody levels in patient sera and correlating these with posttransplant HCMV viremia. Vaccination of seropositive patients significantly boosted preexisting antibody levels against the immunodominant region AD1 as well as against AD2, AD4, and AD5. A decreased incidence of viremia correlated with higher antibody levels against AD2 but not with antibody levels against the other 3 ADs. Overall, these data support the hypothesis that antibodies against AD2 are a major component of the immune protection of seropositives seen following vaccination with gB/MF59 vaccine and identify a correlate of protective immunity in allograft patients.

## Teaser

Human cytomegalovirus (HCMV) glycoprotein B (gB) generates a prodigious polyclonal antibody response in vivo. Patients with antibodies that detect antigenic domain 2 display reduced viraemia post transplant - levels of which are boosted in recipients of a recombinant gB vaccine.

**(See the Editorial commentary by Schleiss, on pages 1861–4.)**

Human cytomegalovirus (HCMV) is a ubiquitous human pathogen [1]. Primary infection is normally asymptomatic in healthy individuals, likely reflecting control of virus replication by the immune system. However, HCMV can be a major cause of morbidity following infection of immunocompromised individuals such as solid organ transplant (SOT) patients, hematopoietic stem cell transplant recipients [2–5], fetuses infected in utero [6, 7] and late-stage AIDS patients [8, 9]. The socioeconomic and clinical burden of CMV infection led the Institute of Medicine to designate development of a HCMV vaccine as the highest priority [10]. The first attempts to vaccinate against HCMV were made with live attenuated Towne and AD169 strains [11, 12] followed by subunit and vectored vaccines reviewed elsewhere [13, 14], but an HCMV vaccine is not yet licensed for clinical use.

The glycoprotein B (gB) protein is highly conserved across the herpesvirus family and is essential for viral entry [15–17]. Neutralizing and function-blocking antibodies (ie, antibodies that bind to an antigen and inhibit its normal function without necessarily destroying the pathogen) targeting gB effectively inhibit HCMV infection in vitro. Early studies speculated that most (40%–70%) of the serum-neutralizing activity against HCMV in vivo is directed towards gB [18]. These estimates were based on neutralization of fibroblast infection largely with laboratory strains, whereas additional complexes are now known to perform cell-type–specific functions in entry (most notably the pentameric complex in nonfibroblast cells) [19]. However, the role of gB in entry into all cell types retains this protein as an attractive target for vaccination.

Support for gB as an attractive vaccine component comes from studies with animal models demonstrating that a recombinant gB vaccine decreased the rate of virus transmission in pregnant guinea pigs and mortality amongst new-born pups [20]. In humans, gB vaccine with MF59 adjuvant (gB/MF59) proved to be safe and immunogenic [21–23], reducing primary infection in adult women by approximately 50% [24], by 42% in adolescent girls, and partially controlling viremia in SOT recipients [25, 26].

Although all 3 phase 2 clinical trials of gB/MF59 provide evidence of a protective effect, the exact correlates of protection remain unclear [25–27]. In the SOT patients the duration of viremia was inversely correlated with the anti-gB antibody titer, suggesting that humoral responses may be protective [25]. The humoral response against gB is polyclonal with 5 major antigenic domains (ADs) identified [28]. The first, highly conserved neutralizing epitope was identified on gp55 of gB using monoclonal antibodies [29]. A defined stretch of amino acids (aa 608–625) was a component of the larger AD1 region, which consists of approximately 80 aa between positions 560 and 640 of gB (gp58) in the AD169 strain [30]. Subsequent homology studies between Towne and AD169 strains revealed AD2 contains 2 binding sites: site I, located between aa 68 and77, is conserved amongst strains and antibodies that bound to this site were neutralizing; site II, located between aa 50 and 54, is unconserved between strains and bound antibodies were incapable of neutralizing the virus [31]. An additional linear epitope, AD3, was mapped to a sequence in the intraluminal part of the gB molecule (between aa 798 to 805) suggesting that this region may not be exposed to neutralizing antibody responses. Most recently, an analysis of the repertoire of gB-specific memory B cells identified 2 structural antibody domains targeted by antibodies with neutralizing activity. These were defined as domain I (AD5, located between aa 133 and 343) and domain II (AD4, a discontinuous domain mapped to aa 121–132 and aa 344–438) [28]. In summary, it is evident that AD1 is a major target of humoral response because nearly 100% of sera from HCMV healthy seropositive donors have antibodies that bind to this antigenic domain [32, 33]. However, because AD1 induces a mixture of neutralizing and nonneutralizing specificities, it was initially suggested that antibodies directed against other domains, such as AD2, may confer better protection against HCMV infection [34]. This possibility requires further evaluation, especially now that AD4 and AD5 have been identified.

In this study we characterized the antibody repertoire against major antigenic domains of gB following natural infection and vaccination with gB/MF59 in the sera from patients who were naturally seropositive prior to vaccination. We report that vaccination boosted preexisting responses but displayed a variable capacity to induce de novo responses against these ADs. Importantly, we provide evidence that responses against the AD2 domain directly correlate with better outcomes posttransplant. Additionally, we provide evidence to suggest AD1 responses, which have been hypothesized to reduce the effectiveness of humoral immunity against HCMV, are not detrimental in this transplant patient cohort. More generally, the data illustrate the complexity of studying the immune response to identify correlates of protection to prevent HCMV viremia and disease.

## MATERIALS AND METHODS

### Antigens

The following gB-specific antigens, derived from HCMV strain AD169, were used: AD1 containing aa 484–650, was expressed with galactosidase as a fusion partner in *Escherichia coli*. The construction of galactosidase-containing plasmids has been described in detail elsewhere [30].

AD2, a short linear peptide containing aa 68–80, was synthesized chemically, as described in detail elsewhere [30, 33].

AD4 contained a fused polypeptide of aa 121–132 and 344–438. For determination of AD4-specific antibodies a purified GST–AD4 fusion protein was used as antigen and expressed in *E. coli,* as described by Spindler et al [35].

AD5 contained aa 133 to 343. AD5-specific antibodies were determined in a capture enzyme-linked immunosorbent assay (ELISA) using a mammalian cell (HEK 293T) derived AD5 polypeptide containing an HA epitope tag at the amino terminus of the protein, as described elsewhere [36]. To capture the antigen, an anti-HA monoclonal antibody (clone HA-7, Sigma-Aldrich) was diluted to 1 µg/mL in 0.05 M sodium carbonate buffer pH 9.6, and 50 µL/well was used to coat polystyrene 96-well plates (NuncImmuno) overnight at 4°C.

### ELISA

All reactions were performed at 37°C. Reaction wells were rinsed with phosphate-buffered saline (PBS) supplemented with 0.1% Tween then the reaction wells were blocked with PBS containing 2% fetal calf serum (FCS) for 1 hour, washed 3 times with PBS plus 0.1% Tween 20 and incubated with antigens for 2 hours. The plate was washed 3 times with PBS containing 0.1% Tween 20 and human serum was added at a dilution of 1:100 for 1 hour. Dilution of all sera was done in PBS with 2% FCS. Unbound antibody was removed by washing 3 times and peroxidase-conjugated secondary antibody (goat-anti-human IgG; Dianova) was added for 1 hour. After 3 washing steps with phosphate-buffered saline (PBS) supplemented with 0.1% Tween, 100 µL of tetramethylbenzidine peroxidase substrate was added for 3.5 minutes, diluted 1:1 in peroxidase substrate solution B (KPL). The reaction was stopped by adding 100 µL of 1 M phosphoric acid. The optical density at 450 nm (OD450) was determined using an Emax microplate reader (Eurofins MWG Operon, Germany).

The cutoff value was calculated based on the 2 standard deviations above the mean of the OD values in ELISA results with sera from seronegative patients (n = 20).

### Patient Population

The population investigated in this work is a subset of the original vaccine cohort (CMV seropositive prevaccination) from a group of SOT patients (NCT00299260) enrolled in a phase 2 randomized and double-blinded placebo-controlled cytomegalovirus glycoprotein-B vaccine with MF59 adjuvant trial [25]. All prospective transplant patients are serotyped as part of UK National Health Service standard procedure using an antibody-based ELISA. The vaccine or placebo was given in 3 doses: at day 0 (baseline), 1 month, and 6 months later. Blood samples were collected at: day 0; at the time of vaccination (visit 1); at the time of the administration of the second dose; 1 month following the administration of the first dose (visit 2); at 2 months following the administration of the first dose (visit 3); at the time of the administration of the third dose; 6 months following the administration of the first dose (visit 4); and at 7 months following the administration of the first dose of vaccine (visit 5). Exclusion criteria included: pregnancy (a negative pregnancy test was required before each vaccine dose); receipt of blood products (except albumin) in the previous 3 months; and simultaneous multiorgan transplantation [25]. The study was approved by the Research Ethics Committee and all patients whose samples were investigated here gave written informed consent [25].

### Samples

Blood samples (5 mL) were collected in sterile tubes (without anticoagulant) and then left in a standing position for approximately half an hour to allow blood to clot. The samples were centrifuged at room temperature at 1500*g* for 15 minutes and the serum fraction separated from the clot. Serum samples were stored at −78°C prior to analysis.

### Statistical Analyses

The analysis of the results was performed by Graph Pad Prism software. Statistical differences between the mean value of the OD of the samples obtained at the same time points in the same experimental run between populations of patients: vaccinated versus placebo and viremia versus no viremia were obtained from Mann-Whitney test (ns, not significant; * *P* < .05; ** *P* < .005; *** *P* < .005). Geometric mean values (±95% confidence interval [CI]) were represented by horizontal lines.

## RESULTS

### Vaccination Boosts Preexisting Immune Responses Against Epitopes of gB but Only Induces Detectable De Novo Responses Against Some Epitopes

**Figure 1.**
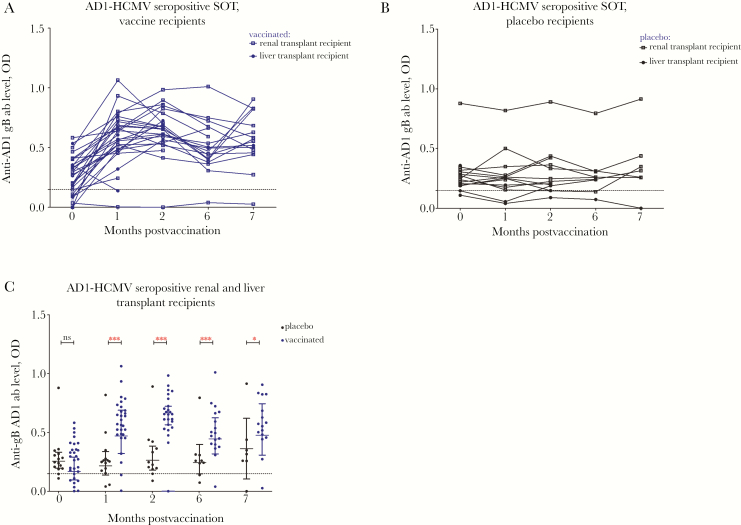
The majority of seropositive patients have preexisting antigenic domain 1 (AD1) immune responses boosted by vaccination. AD1 responses are represented as optical density (OD) values at different time points: day of first vaccine/placebo administration (month 0); day of administration of the second (month 1) and third dose (month 6); and 2 and 7 months postvaccination. *A*, AD1 responses in human cytomegalovirus (HCMV) seropositive vaccine recipients represented as OD values. *B*, AD1 responses in HCMV seropositive placebo recipients represented as OD values. *C*, Comparison between antibody levels against AD1 in the sera from vaccinated and placebo patients. Horizontal lines represent geometric mean values (± 95% confidence interval). Statistical differences between the mean value of ODs between the populations of patients: vaccinated versus placebo were obtained from Mann-Whitney test (ns, not significant; * *P* < .05; ** *P* < .005; *** *P* < .005). Abbreviation: SOT, solid organ transplant.

**Figure 2.**
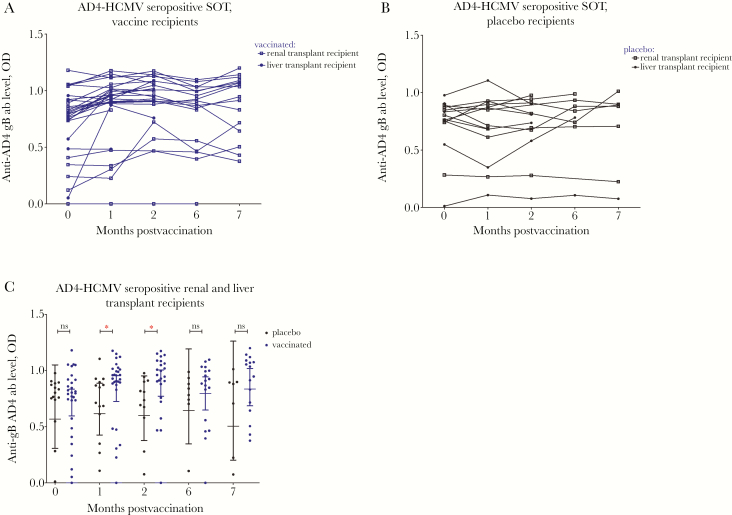
The majority of seropositive patients have preexisting antigenic domain 4 (AD4) immune responses boosted by vaccination. AD4 responses are represented as optical density (OD) values at different time points: day of first vaccine/placebo administration (month 0); day of administration of the second (month 1) and third dose (month 6); and 2 and 7 months postvaccination. *A*, AD4 responses in human cytomegalovirus (HCMV) seropositive vaccine recipients represented as OD values. *B*, AD4 responses in HCMV seropositive placebo recipients represented as OD values. *C*, Comparison between antibody levels against AD4 in the sera from vaccinated and placebo patients. Horizontal lines represent geometric mean values (± 95% confidence interval). Statistical differences between the mean value of ODs between the populations of patients vaccinated versus placebo were obtained from Mann-Whitney test (ns, not significant; * *P* < .05). Abbreviation: SOT, solid organ transplant.

**Figure 3.**
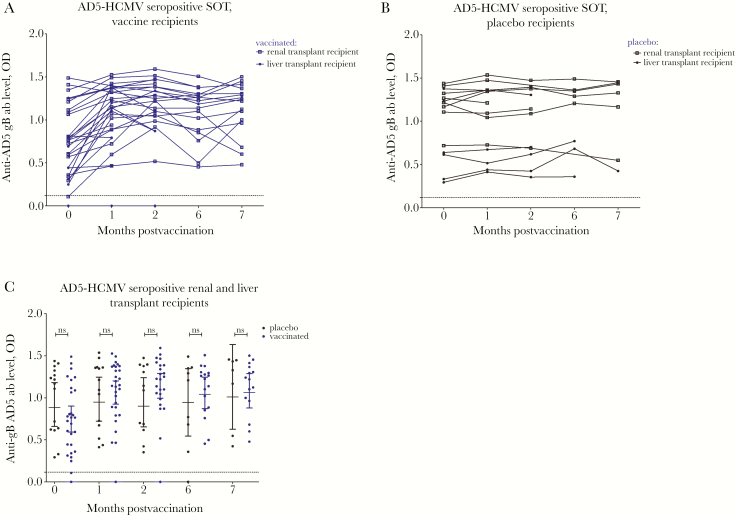
Vaccination boosts preexisting antigenic domain 5 (AD5) responses and induces detectable de novo responses in patients. AD5 responses are represented as optical density (OD) values at different time points: day of first vaccine/placebo administration (month 0); day of administration of the second (month 1) and third dose (month 6); and 2 and 7 months postvaccination. *A*, AD5 responses in human cytomegalovirus (HCMV) seropositive vaccine recipients represented as OD values. *B*, AD5 responses in HCMV seropositive placebo recipients represented as OD values. *C*, Comparison between antibody levels against AD5 in the sera from vaccinated and placebo patients. Horizontal lines represent geometric mean values (± 95% confidence interval). Statistical differences between the mean value of ODs between the populations of patients vaccinated versus placebo were obtained from Mann-Whitney test (ns, not significant). Abbreviation: SOT, solid organ transplant.

**Figure 4.**
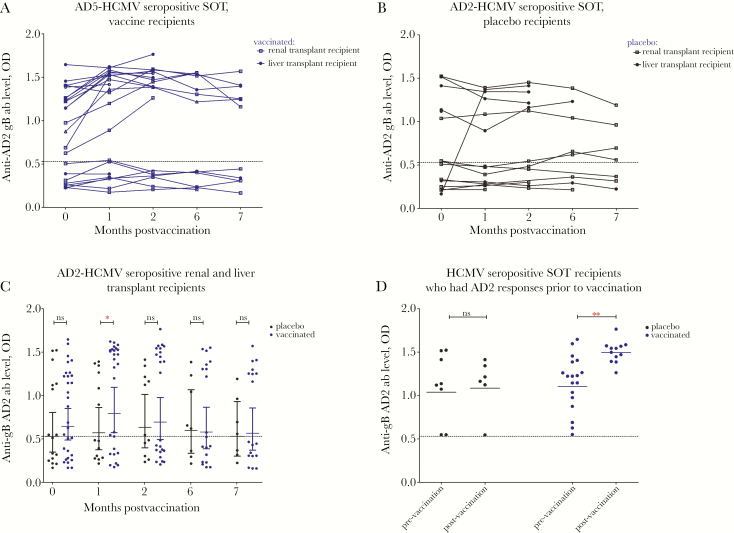
Vaccination does not induce detectable de novo responses in patients lacking preexisting antigenic domain 2 (AD2) responses but boosts preexisting antibody responses above cutoff against AD2 in human cytomegalovirus (HCMV) seropositive patients. AD2 responses are represented as optical density (OD) values at different time points: day of first vaccine/placebo administration (month 0); day of administration of the second (month 1) and third dose (month 6); and 2 and 7 months postvaccination. *A*, AD2 responses in HCMV seropositive vaccine recipients represented as OD values. *B*, AD2 responses in HCMV seropositive placebo recipients represented as OD values. *C*, Comparison between antibody levels against AD2 in the sera from vaccinated and placebo patients. *D*, Comparison between antibody levels against AD2 responses in patients who had preexisting antibody responses. AD2 responses are represented as OD values at day of first vaccine/placebo administration (prevaccination) and 2 months following the administration of the first dose of the vaccine (postvaccination). The dotted line represents a cutoff value (the highest OD value in seronegative group at the time of vaccine administration). Horizontal lines represent geometric mean values (± 95% confidence interval). Statistical differences between the mean value of ODs between the populations of patients vaccinated versus placebo were obtained from Mann-Whitney test (ns, not significant; * *P* < .05; ** *P* < .005). Abbreviation: SOT, solid organ transplant.

To investigate serological responses we utilized ELISA assays against 4 key antigenic domains of gB: AD1, 2, 4, and 5 (Figures 1–4). Specific antibody responses were measured at 5 different time points (visits 1–5): day of vaccine/placebo administration (month 0, visit 1); day of administration of the second dose (month 1, visit 2) and third dose (month 6; visit 4); and 2 months (visit 3) and 7 months postvaccination (visit 5) (summarized in Supplementary Table S1).

To establish the background values for each antigenic domain we utilized sera from seronegative SOT patients collected at the time of their vaccine or placebo administration. We used the highest values detected in those seronegative individuals to establish cutoff points.

The data show that nearly all the HCMV seropositive individuals possessed detectable antibodies against AD1 (Figure 1A and 1B). Vaccination increased preexisting antibody levels against AD1 in nearly all individuals (Figure 1A and 1C; Table 1). This boost was observed by dose 1 and subsequently sustained at increased levels up to the time of transplantation.

**Table 1.** Summary of Antibody Responses in Sera From Human Cytomegalovirus (HCMV) Seropositive Patients Vaccinated with the Glycoprotein B Subunit (gB) Vaccine with MF-59 Adjuvant Against 4 Key Antigenic Domains Mapped onto gB

Antigenic Domain	HCMV Seropositive Vaccine Recipients				
	Induction of Antibody Responses De Novo	Boost of Preexisting Responses	Positivity Prior to Vaccination, % (No. Positive/Total)	Positivity Following Vaccination, % (No. Positive/Total)	Protection From Viremia
AD1	Yes (Figure 1)	Yes (Figure 1)	86.4% (38/44)	93.8% (15/16)	No
AD2	No (Figures 4 and 5)	Yes (Figures 4 and 5)	50% (23/46)	50% (9/18)	Yes
AD4	No (Figure 2)	Yes (Figure 2)	98% (43/44)	93.8% (15/16)	Trend
AD5	Yes (Figure 3)	Yes (Figure 3)	97.7% (43/44)	95.8% (23/24)	No

Protection from viremia is defined as when patient did not experience an episode of viremia during the course of analyses (viremia>200 cps/mL).

Similar results were observed with AD4 (Figure 2A and 2B; Table 1). In seropositive patients with low-level baseline AD4 antibody responses we observed increased anti-AD4 antibody levels postvaccination in some, but not all, individuals (Figure 2A and 2C).

Sera from nearly all patients contained antibodies recognizing AD5 (Figure 3A and 3B; Table 1). Vaccination increased preexisting antibody levels against AD5 in the majority of patients (Figure 3A and 3C). In the few patients with AD5 levels below the cutoff value (by ELISA) prior to vaccination we saw evidence that vaccination promoted de novo responses in some of these patients also.

Approximately 50% of patients had levels of anti-AD2 antibodies above the background cutoff value prior to vaccination (Figure 4A and 4B; Table 1). Administration of the first dose of gB/MF59 was sufficient to boost preexisting antibody levels against AD2 in most HCMV seropositive SOT patients (Figure 4A and 4C). When the analysis was restricted to those with the levels of AD2 antibodies above the background cutoff at baseline, it became clear that this boost was statistically significant (Figure 4D).

### Higher AD2 Antibody Levels Correlate With Lower Incidence of Viremia Posttransplantation

**Figure 5.**
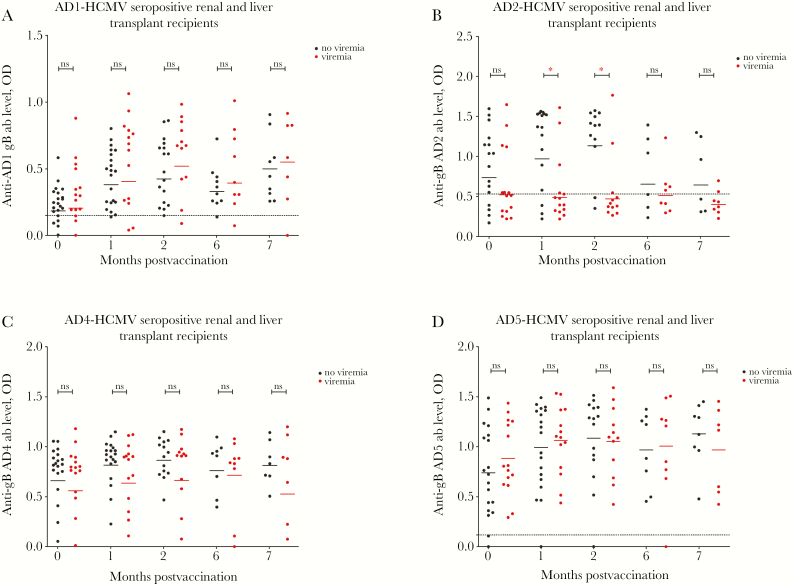
Elevated antibody responses against antigenic domain 2 (AD2) correlate with protection. Comparison of antibody levels against AD1 (*A*), AD2 (*B*), AD4 (*C*), and AD5 (*D*) between patients who developed viremia versus patients who did not develop viremia following transplantation at day of first vaccine/placebo administration (month 0); day of administration of the second (month 1) and third dose (month 6); and also at times 2 and 7 months post initial vaccination. Horizontal lines represent geometric mean values. Statistical differences between the mean value of optical densities (ODs) between the populations of patients viremia versus no viremia were obtained from Mann-Whitney test (ns, not significant; * *P* < .05).

We next investigated the correlation between antibody levels against specific ADs and outcome posttransplantation (Figure 5). Despite clear evidence of a boost in responses to AD1, AD4, and AD5 (Figures 1–3), there was no statistically significant correlation with the occurrence of viremia among the patients who underwent transplantation (Figure 5A, 5C, and 5D). However, we note that in the case of AD4 a nonsignificant trend was evident, whereby patients who had higher levels of AD4-specific antibody responses were less likely to develop viremia (Figure 5C).

In contrast, it was clear that the AD2 antibody level was significantly lower in the patients who developed viremia following transplant, consistent with the hypothesis that antibodies against AD2 are protective (Figure 5B). This protection was restricted to patients with AD2 responses prior to vaccination because vaccination itself did not induce detectable AD2 responses de novo (Figure 4).

### The Correlation With Protection Observed With AD2 is Not Affected by AD1 Responses

**Figure 6.**
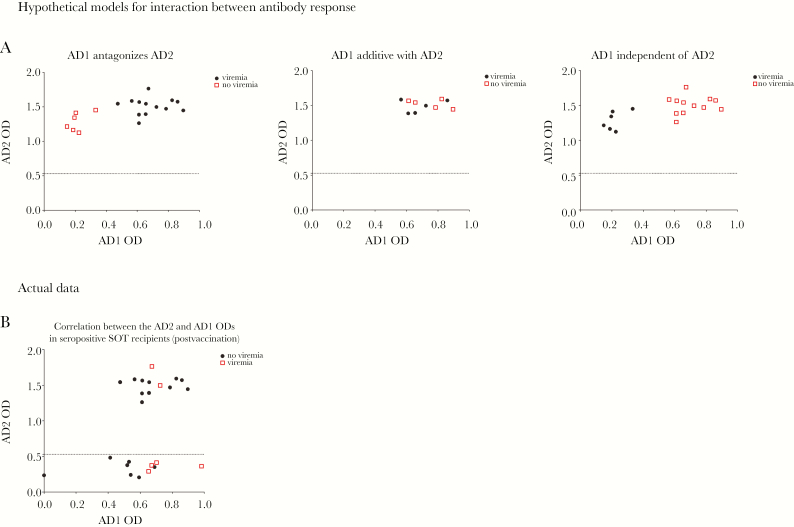
No evidence for antagonistic antibody responses between antigenic domain 1 (AD1) and AD2 affecting outcome. *A*, Hypothetical models of interactions between AD1 and AD2 antibody responses and clinical outcome. *B*, AD2 (Y axis) and AD1 (X axis), represented as optical density (OD) values at 2 months following the administration of the first dose of vaccine. Squares represent patients who subsequently developed viremia posttransplant and circles represent patients who did not experience viremia posttransplant.

We next asked whether these data could test for interactions between the antibody responses. Underpinning this approach is a prior hypothesis that AD1 responses may negatively impact on AD2 responses [37]. Theoretically, there are 3 possible relationships between the AD1 and AD2 antibody levels in vaccinated seropositive SOT recipients and their effect on outcome: (1) competition (promoting viremia); (2) additive effect (promoting protection); and (3) no direct interaction (Figure 6A).

To address this, we performed a 2-component analysis where patient sera were stratified for outcome (viremia versus no viremia) and then both AD1 and AD2 responses plotted. The resulting graph demonstrates no correlation between the AD2 and AD1 levels in seropositive SOT recipients postvaccination that segregated with viremia (Figure 6B). However, attempts to explore this further using multivariable statistical analysis were not possible because the clinical trial population size provided insufficient data points for more complex analyses (results not shown).

## DISCUSSION

This work illustrates the complexity of studying immune responses to HCMV in seropositives. For example, HCMV establishes latency from which it periodically reactivates, which could alter the pattern of immunological responses seen at any time of analysis irrespective of any external vaccine administration. To control for this, we examined not only vaccinated patients but also seropositive recipients of placebo at the same time points. This allowed us to follow natural changes in the composition of the humoral immune response in seropositive transplant candidates who experienced a virus challenge at the time of transplantation. Here we aimed to provide more insight into the protective nature and fine specificity of the humoral responses against gB. To be classified as a correlate of protection following vaccination, any immunological responses would need to be induced or boosted by the vaccine and to correlate with protection against posttransplant viremia [25].

A major observation in this study was boosting of preexisting responses to all 4 antigenic domains by gB/MF59. However, only antibody titers against AD2 correlated with protection against viremia. This illustrates that, for vaccine development, demonstrating immunogenicity is not sufficient and requires supplementation with studies of protection in human challenge models, such as that employed here. We demonstrated that AD2 antibody levels displayed the strongest correlation with protection in our seropositive patient cohort. However, the vaccine was not observed to induce de novo AD2 responses, but boosted preexisting responses. Previous studies have shown approximately 50% of infected individuals possess antibodies against site I of AD2 following natural infection [31–33, 38] and the data we present here are consistent with this. Recent structural and immunochemical analyses suggest that the anti-AD2–specific immunological responses may be created though a cascade of rare and very specific immunoglobulin gene rearrangement events [34, 39]. Possibly, therefore, the variable response towards this epitope following both natural infection and vaccination with gB/MF59 and Towne-based vaccines is a consequence of the low probability of developing antibodies that require recombination of 1 of 2 well-conserved human germline V elements (IGHV3-30 and IGKV3-11) and IGHJ4, and the possibility of antigen competition through the simpler production of AD1 antibodies [37]. Antibodies against AD2 are also characterized by specific substitutions at certain positions that seem to be crucial for high-affinity binding to this epitope [34, 40–42]. Although only a proportion of infected individuals develop these AD2 antibodies, they may contribute an important neutralizing activity for controlling infection [38, 42–44]. Thus an immunogen that can enhance or generate de novo responses against AD-2 may be a good candidate for a new HCMV vaccine. It is important to reiterate that our data suggest that, whilst preexisting AD2 responses established at the time of primary infection or reactivation of the virus from latency can be enhanced, the gB/MF59 vaccine does not induce detectable AD2 responses in those lacking them at baseline. However, the study did reveal a marked increase in AD2 levels in 1 recipient of placebo. We hypothesize that this might be a response to reactivation of latent virus or even a reinfection event in this patient prior to transplant, illustrating how responses may develop over time. Although these data support a role for AD2 antibodies in the control of HCMV infection, other components of the humoral response could be important as well, including AD4, which deserves further investigation. In vitro studies show that AD4 specific antibodies have a high neutralizing capacity at the postadsorption step [28]. Indeed, antibodies that bind to the AD4 corresponding sequence on HSV-gB inhibit the interaction of gB with gH/gL complex with a downstream effect on viral fusion [45]. Antibodies that impeded this aspect of viral entry could potentially impact on viral infection. Although the AD4 association did not reach statistical significance, this could be due to the number of patients available to us. Serological analysis of this vaccine cohort revealed that the AD1 and AD5 antibody levels did not correlate with protection. The humoral response to natural infection against the immunodominant region AD1 has variable neutralizing capacity [46]. Competition between nonneutralizing and neutralizing antibodies against AD1 was reported [18, 29, 46], suggesting that AD1 antibody binding may even provide an immune-evasive mechanism by preventing the binding of other neutralizing antibodies to cell-free virus [46]. It is tempting to speculate whether AD1 should be removed from HCMV subunit vaccines. If the elimination of AD1 improved antibody responses against protective epitopes this would support such a modification (as has been proposed for AD2) although we could find no evidence in our cohort to support this hypothesis. Additionally, we cannot rule out that AD1 provides key structural information ensuring the better presentation of “good” epitopes. Indeed, attempts to engineer gB without AD1 have proven difficult as AD1 is necessary for oligomerization and the structural integrity of gB [47]. This lack of structural information may explain a preclinical study that demonstrated a peptide-based vaccine specific to the HCMV gB AD2 region elicited only poor neutralizing antibody responses [48]. However, we also emphasize that we have previously reported [25] that protection given by this vaccine did not correlate with neutralizing activity. This is not to disregard neutralization as a strategy because preclinical studies with monoclonal anti-AD2 antibody (TRL345) have shown promising results, supporting its further investigation as a candidate for clinical evaluation [49].

Although our analyses of the AD5 humoral response did not reveal a protective correlation it did reveal some interesting information regarding the response to this antigen [28, 36]. First reports of AD5 immunogenicity suggested approximately 50% of seropositive individuals developed AD5 antibodies [28]. However, using second-generation antigens and tests, seropositivity rates in healthy HCMV-infected individuals have been suggested to be in the range of 90% (A. Wiegers and M. Mach, unpublished results) and the data presented here support this.

It is also important to reiterate that OD values that are in the range of background are not necessarily indicative that a serum lacks antibodies to these epitopes. First, genuine epitope-specific antibodies could be potentially present at very low levels not detectable by ELISA. Therefore a significant boost of these antibodies after just 1 vaccine dose could be explained by the existence of a memory B-cell response specific to these epitopes. Alternatively, we cannot rule out the presence of some antibodies that react to the epitope in the context of native gB but fail to react in the ELISA because the epitope is not in its fully native context when presented as a partial subdomain of gB.

Finally, although the data suggest AD2 levels are an important correlate of protection we do not rule out the possibility that responses against other, potentially novel, epitopes may also contribute. Attempts to perform a multivariable analysis to test this were not possible due to the limited number of patients in the study (as the number of variables increases so does the requirement for more patients). Thus future phase 2 studies may need to be powered to ensure sufficient patients are recruited to allow more complex multivariate analyses. Future studies should also ensure the repeated sampling of the patients about to be challenged with the virus at the time of transplantation, the use of a randomized study design, and incorporation of placebo controls — all aspects we consider significant strengths of our study.

Overall, the results described in this work build upon previous reports and support the concept that vaccination should be studied as a way of controlling HCMV replication. Although this analysis gives us more insight into the protective nature of humoral responses elicited by vaccination in seropositive SOT patients, many questions remain unanswered and follow-up phase 2 studies with larger numbers of subjects recruited would add weight to all our observations. Additional antibody-mediated effects may be important for the protection observed (eg, complement-mediated cell lysis and natural killer antibody-dependent cell-mediated cytotoxicity) and this is the subject of ongoing investigation in the quest to provide protection against this important human pathogen.

## Supplementary Data

Supplementary materials are available at *The Journal of Infectious Diseases* online. Consisting of data provided by the authors to benefit the reader, the posted materials are not copyedited and are the sole responsibility of the authors, so questions or comments should be addressed to the corresponding author.

Supplementary Table S1

## Notes

***Disclaimer***. Funding source (VacTrain) had no role in the study design, data collection, data analysis, data interpretation, writing, or in the decision to submit to publication.

***Financial support.*** This work was supported by the European Union under the FP7 Marie Curie Action VacTrain (grant number 316655) and Deutsche Forschungsgemeinschaft (grant number MA 929/11-1). M. B. R. was supported by a Medical Research Council Fellowship (grant number G:0900466). The original clinical trial of gB/MF59 was supported by the National Institute of Allergy and Infectious Diseases (grant number R01AI051355) and Sanofi Pasteur.

***Potential conflicts of interest.*** S. P. and F. D. P. are employees of Sanofi Pasteur. All authors have submitted the ICMJE Form for Disclosure of Potential Conflicts of Interest. Conflicts that the editors consider relevant to the content of the manuscript have been disclosed.

Presented in part: 41st Annual International Herpesvirus Workshop, Madison, Wisconsin, 23–27 July 2016; 11th Mini-Herpesvirus Workshop Berlin, Germany, 30 September 2016; 3rd UK CMV Conference, Cardiff, Wales, 24–25 November 2016; 6th International Congenital CMV Conference/16th International CMV/Betaherpesvirus Workshop, Noordwijkhout, Netherlands , 2017.
