# Supplementary material for: Epitope-Specific Humoral Responses to Human Cytomegalovirus Glycoprotein-B Vaccine With MF59: Anti-AD2 Levels Correlate With Protection From Viremia
Source: J Infect Dis. 2018 Mar 8;217(12):1907–17. doi: 10.1093/infdis/jiy102 (PMC5972559; doi:10.1093/infdis/jiy102)
Supplement: Supplementary Table S1 [file jiy102_suppl_supplementary-table.docx]

|  | **Placebo** | **Vaccinated** |
| --- | --- | --- |
| Number | 15 | 29 |
| **Ever had viremia>200 cps/ml** |  |  |
| Yes | 6 (40.0%) | 8 (27.6%) |
| No | 4 (26.7%) | 10 (34.5%) |
| N/a | 5 (33.3%) | 11 (37.9%) |
| **Ever had viremia>3000 cps/ml** |  |  |
| Yes | 2 (13.3%) | 1 (3.5%) |
| No | 8 (53.3%) | 17 (58.6%) |
| N/a | 5 (33.3%) | 11 (37.9%) |
| **Have result available at** |  |  |
| Visit 1  Day of vaccine/placebo  administration | 15 (100.0%) | 29 (100.0%) |
| Visit 2  Administration of the 2nd dose of vaccine/placebo  (1 month post vaccination) | 14 (93.3%) | 29 (100.0%) |
| Visit 3  (2 months post vaccination) | 12 (80.0%) | 24 (82.8%) |
| Visit 4  Administration of the 3^rd^ dose of vaccine/placebo  (6 months post vaccination) | 9 (60.0%) | 19 (65.5%) |
| Visit 5  (7 months post vaccination) | 7 (46.7%) | 16 (55.2%) |

* N/A means no data available due to not proceeding to transplant or withdrawing from study, therefore their viraemia/no viraemia status remains unknown
